# San-Huang-Xie-Xin-Tang Prevents Rat Hearts from Ischemia/Reperfusion-Induced Apoptosis through eNOS and MAPK Pathways

**DOI:** 10.1093/ecam/neq061

**Published:** 2011-04-14

**Authors:** Shu-Fen Liou, Hung-Jen Ke, Jong-Hau Hsu, Jyh-Chong Liang, Hung-Hong Lin, Ing-Jun Chen, Jwu-Lai Yeh

**Affiliations:** ^1^Department of Pharmacy, Chia-Nan University of Pharmacy and Science, Tainan, Taiwan; ^2^Department and Graduate Institute of Pharmacology, Kaohsiung Medical University, Kaohsiung 807, Taiwan; ^3^Department of Paediatrics, Faculty of Medicine, College of Medicine, Kaohsiung Medical University, Taiwan; ^4^Department of Paediatrics, Kaohsiung Medical University Hospital, Kaohsiung, Taiwan; ^5^Graduate Institute of Engineering, National Taiwan University of Science and Technology, Taipei, Taiwan

## Abstract

San-Huang-Xie-Xin-Tang (SHXT) is a traditional Chinese medication consisting of three herbs, namely *Coptidis rhizome*, *Scutellariae radix* and *Rhei rhizome*. This study aimed to examine the cardioprotective effects of SHXT in a rat model of acute myocardial apoptosis induced by ischemia/reperfusion (I/R). Vehicle (intravenous saline) or SHXT (intravenous or oral) was administered prior to I/R (occlusion of left coronary artery for 45 min followed by reperfusion for 2 h). In the vehicle group, myocardial I/R caused myocardial infarction with increased plasma cardiac enzymes, severe arrhythmia and mortality. Myocardial apoptosis was induced by I/R as evidenced by DNA ladder and Bcl-2/Bax ratio. In the SHXT group, we found that SHXT significantly reduced plasma levels of cardiac enzymes, arrhythmia scores (from 5 ± 1 to 2 ± 1, *P* < .01) and mortality rate (from 53 to 0%, *P* < .01). In addition, pretreatment with intravenous SHXT reduced the infarct size dose-dependently when compared with the vehicle group (10 mg kg^−1^: 14.0 ± 0.2 versus 44.5 ± 5.0%, and 30 mg kg^−1^: 6.2 ± 1.2% versus 44.5 ± 5.0%, both *P* < .01). Similarly, oral administration of SHXT reduced the infarct size dose-dependently. Furthermore, SHXT markedly decreased the apoptosis induced by I/R with increased Bcl-2/Bax ratio. Finally, we found that SHXT counteracted the I/R-induced downstream signaling, resulting in increased myocardial eNOS expression and plasma nitrite, and decreased activation of ERK1/2, p38 and JNK. These data suggest that SHXT has cardioprotective effects against I/R-induced apoptosis, and that these effects are mediated, at least in part, by eNOS and MAPK pathways.

## 1. Introduction

Myocardial injury from ischemia/reperfusion (I/R) is a clinical problem requiring invasive interventions such as thrombolysis, angioplasty and coronary bypass surgery, to reestablish coronary blood flow and minimize cardiac damage due to severe myocardial ischemia [1]. Reperfusion of an occluded coronary artery is known to reduce infarct size, preserve left ventricular function, and reduce overall mortality. However, it is now recognized that the readmission of oxygenated blood into previously ischemic myocardium can initiate a cascade of events that will paradoxically produce additional myocardial cell dysfunction and cell apoptosis [1–3].

San-Huang-Xie-Xin-Tang (SHXT), a widely used traditional Chinese medication, consists of three herbs, namely *Coptidis rhizome* (*Coptis chinesis* Franch), *Scutellariae radix* (*Scutellaria baicalensis* Georgi) and *Rhei rhizome* (*Rheum officinale* Baill). Previous laboratory and clinical studies have suggested that SHXT may have a role in the treatment of various diseases including gastrointestinal disorders [4], acute lung injury [5], septic shock [6], hypertension [7, 8] and neuronal injury [9]. There are three major bioactive constituents in SHXT: berberine, baicalin and baicalein [10, 11]. Berberine, one of the main components in *Coptidis rhizoma*, has been shown to improve cardiac performance and decrease ventricular premature complexes and mortality in patients with congestive heart failure [12, 13]. In addition, it was recently found that berberine can reduce I/R injury and attenuate I/R-induced apoptosis in myocytes [14]. Baicalin and baicalein are both flavonoids derived from the root of *Scutellaria baicalensis*, and have been reported to protect rat cardiomyocytes against hypoxia/reoxygenation and I/R, partly due to their scavenging capacity for reactive oxygen species (ROS) generated during these insults [15, 16]. Furthermore, *Rhei rhizoma* extracts reportedly possess antioxidant and anti-lipid peroxidation activities through their direct suppression of mitochondrial ROS generation [17].

Even though the individual effects of these three major bioactive constituents of SHXT have been found to be beneficial in the settings of congestive heart failure or I/R-induced myocardial damage [12–16], no studies have examined whether the whole compound of SHXT also conveys cardioprotective effects. Thus, the present study aimed to investigate whether pretreatment with SHXT protects rat hearts against I/R-induced myocardial apoptosis, and if so, whether the anti-apoptotic effects are mediated by nitric oxide (NO) and mitogen-activation protein kinase (MAPK) pathways.

## 2. Methods

### 2.1. Materials and Reagents

The voucher specimens and methods of extraction and analysis of SHXT were the same as we have previously described [5]. Reagents used in this study included: antibodies of eNOS and *β*-actin from Sigma-Aldrich Inc. (St. Louis, MO, USA); antibodies of Bcl-2, Bax, ERK1/2, phosphorylated ERK1/2, phosphorylated p38, JNK and phosphorylated JNK from Upstate Biotechnology (Lake Placid, NY, USA); antibody of p38 from Santa Cruz Biotech (Santa Cruz, CA, USA); PVDF membrane and an enhanced chemiluminescence (ECL) kit from PerkinElmer Life and Analytical Sciences (Waltham, MA, USA), and peroxidase-conjugated immunoglobulins from Calbiochem (Temecula, CA, USA).

### 2.2. Experimental Animals

Wistar rats from the National Laboratory Animal Breeding and Research Center (Taipei, Taiwan) were housed under conditions of constant temperature and controlled illumination (light on between 7:30 and 19:30 hours). Food and water were available ad libitum. The study was approved by the Animal Care and Use Committee of the Kaohsiung Medical University.

### 2.3. Experimental Protocol

The I/R protocol was performed as we have previously described [18]. In brief, male Wistar rats weighing 250–300 g were anesthetized with intraperitoneal pentobarbital sodium (40 mg kg^−1^). Tracheotomy was performed and an intubating cannula was connected to a rodent ventilator. The animals were ventilated artificially with room air. The left femoral artery and vein were cannulated for the measurement of arterial blood pressure (ABP) and heart rate (HR) via a Statham pressure transducer and a Biotechnometer (AD Instruments, Mountain View, CA, USA), and for the administration of drugs, respectively. After a left-side thoracotomy was performed at the fourth intercostal space, the pericardium was incised and the heart was exteriorized. A ligature (6/0 silk suture) was placed around the left main coronary artery close to its origin. The thread was then made into a knot as an occluder and another thread was tied to the first knot as a releaser. The ends of both threads were brought outside the thoracic cavity. Thus, the occlusion could be tightened or loosened by pulling the thread of the releaser. The coronary artery was occluded for 45 min, followed by 120 min of reperfusion. Sham-operated animals (sham group) underwent all of the above-described surgical procedures except that the 6/0 silk was passed around the left coronary artery, but was not tied.

SHXT was prepared according to the method we have described previously [5]. Animals were randomized into the following groups: (i) sham, (ii) operated + vehicle (i.v. saline), (iii) operated + SHXT (10 mg kg^−1^, i.v. bolus; 15 min prior to ischemia), (iv) SHXT (30 mg kg^−1^, i.v. bolus), (v) SHXT (10 mg kg^−1^, oral; 1 h prior to ischemia), (vi) SHXT (30 mg kg^−1^, oral).

### 2.4. Evaluation of Arrhythmia and Mortality

For all the groups, heart rate was measured from the recordings of an electrocardiogram (lead II) and the incidence of arrhythmia registered in accordance with the Lambeth Conventions [19] as ventricular tachycardia (VT), ventricular fibrillation (VF) and ventricular ectopic beat (VEB). VEB is defined as a discrete and identifiable premature QRS complex. VT was diagnosed as four or more consecutive VEBs. VF was diagnosed when the ECG recording showed chaotic activity with an amplitude less than that of the normal ECG. Complex forms (e.g. bigeminy) were included in the count of VEB and not analyzed separately. VF may be sustained or may revert spontaneously to a normal sinus rhythm in rats. Irreversible VF was defined as VF which did not reverse within 5 min of onset. The onset and duration of arrhythmia were also measured. The arrhythmia score for these experiments was calculated using a previously published scale [20]. The mortality rate was also recorded in each group.

### 2.5. Determination of Myocardial Infarct Size

At the end of the experiment the coronary artery was occluded and 0.5 mL of 5% Evans blue was injected intravenously to visualize the area at risk of infarction (AAR). The heart was then excised and the atria were removed. The entire ventricular area was sectioned into 2- to 3-mm thick slices from the apex to the base and incubated in 1% triphenyltetrazolium chloride (TTC) for 15 min at 37°C. TTC stained the viable tissue dark red while the infarcted tissue remained grayish-white. The AAR and area of infarct were both traced by hand on an imaging system. The traced areas were then quantified by computerized planimetry [18].

### 2.6. Measurement of Plasma LDH, CK-MB and Troponin I

Myocardial cellular damage and necrosis were evaluated by measuring plasma levels of three cardiac enzymes: lactate dehydrogenase (LDH), troponin I and creatine kinase (CK)-MB. The blood samples were drawn from the femoral artery at the end of reperfusion and collected in heparinized tubes. Samples were promptly centrifuged at 2000 g for 15 min until measurements. Plasma levels of LDH and CK-MB were measured with a commercial kit from Sigma-Aldrich Inc. Serum troponin I was measured with a commercial third-generation electrochemiluminescence immunoassay kit (Roche Diagnostics, Indianapolis, IN, USA).

### 2.7. Determination of Plasma Nitrite Production

Blood samples were collected at the end of the experiment. Samples were promptly centrifuged at 2000 g for 20 min at 4°C. Plasma supernatant was stored at −80°C until analyzed. NO production was spectrophotometrically determined by assaying the plasma supernatant for nitrite using the Griess reagent (1% sulfanilamide and 0.1% naphthylethylenediamide in 5% phosphoric acid). Absorbance was measured at 540 nm and nitrite concentration was determined using sodium nitrite as a standard.

### 2.8. Detection of DNA Fragmentation

Myocardial apoptosis was qualitatively analyzed by detection of DNA fragmentation. Genomic DNA from fresh-frozen tissue was isolated using a Purgene DNA Isolation Kit (Purgene, Minneapolis, MN, USA) according to the manufacturer's instructions. Twenty-five micrograms of DNA was loaded onto 2% agarose gel containing 0.5 *μ*g mL^−1^ ethidium bromide. DNA electrophoresis was carried out at 50 V for 1-2 h. DNA ladder formation, a hallmark of tissue apoptosis, was visualized under ultraviolet light and photographed for a permanent record.

### 2.9. Western Blotting

Left ventricles were removed at the end of the experiment, and lysed with lysis buffer [20 mM Tris-HCl (pH 7.5), 1 mM dithiothreitol (DTT), 5 mM EGTA, 2 mM EDTA, 0.5 mM PMSF, 20 *μ*M leupeptin and 20 *μ*M aprotinin]. After sonication, the lysates were centrifuged and the proteins were separated by electrophoresis on SDS-PAGE (10–14%) and transferred onto a polyvinylidene difluoride (PVDF)-plus membrane. After blocking for 1 h in 5% non-fat dry milk in Tris-buffered saline, the membrane was incubated with the desired primary antibody for 2 h. The membrane was then treated with appropriate horseradish peroxidase conjugated secondary antibody (diluted 1 : 1000), and the immunoreactive bands were detected with enhanced chemiluminescence reagents (PerkinElmer Life and Analytical Sciences).

### 2.10. Statistical Analysis

The results are expressed as mean ± SEM. Statistical differences were estimated by one-way analysis of variance (ANOVA) followed by Dunnett's test. A value of *P* < .05 was considered significant.

## 3. Results

### 3.1. Ventricular Arrhythmia and Mortality Rate

SHXT produced markedly antiarrhythmic effects in anesthetized rats as shown in Table 1. I/R caused pronounced arrhythmogenic activity with 100% VT and 63% VF. In both oral (10 mg kg^−1^) and i.v. bolus SHXT (10 mg kg^−1^) treated groups, there were significant decreases in the number of VEBs (from 781 ± 49 to 535 ± 35 and 326 ± 54, resp., *P* < .05) and the incidence of VF (both from 63 to 25%) and irreversible VF (both from 13 to 0%). Similarly, both oral (30 mg kg^−1^) and i.v. bolus administration of SHXT (30 mg kg^−1^) significantly reduced the total number of VEBs (from 781 ± 49 to 210 ± 30 and 166 ± 28, resp., *P* < .01) and the incidence of VT (both from 100 to 75%), VF (both from 63 to 25%) and irreversible VF (both from 13 to 0%). Moreover, the duration of VT and VF was also significantly reduced in all SHXT-treated groups except for the group with low dose oral administration (10 mg kg^−1^). The most marked reductions in the arrhythmia scores were observed in high dose (30 mg kg^−1^) groups with oral and i.v. bolus administration (both from 5 ± 1 to 2 ± 1, *P* < .01). More importantly, SHXT decreased the mortality rate from 53 to 0% (*P* < .01).

### 3.2. Myocardial Infarct Size

**Figure 1:**
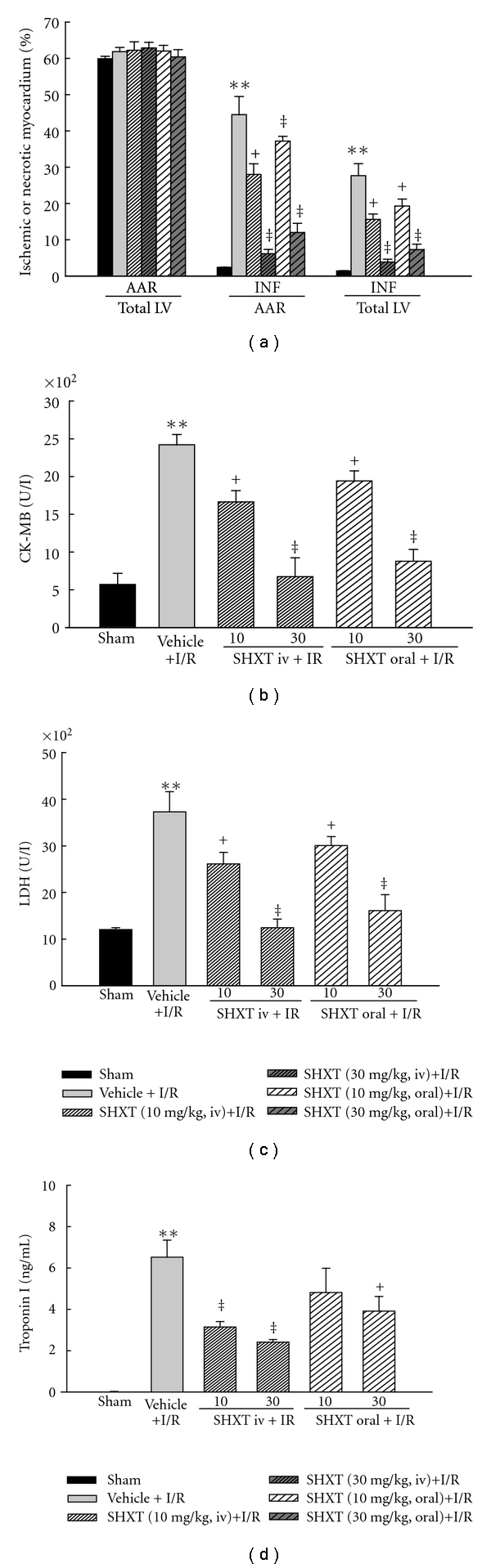
Effects of SHXT on myocardial infarct size and plasma levels of CK-MB, LDH and troponin I in rat hearts subjected to I/R. (a) Bar graphs show area at risk of infarction indexed to total left ventricle (AAR/Total LV), infarcted area indexed to area at risk of (INF/AAR), and infarcted area indexed to total left ventricle (INF/Total LV). (b)–(d) Effects of pretreatment with SHXT on plasma levels of cardiac enzymes. Each value represents the mean ± SEM of six rats. **P < .01 versus sham group; ^+^ *P* < .05, ^‡^ *P* < .01 versus vehicle-treated rats. ANOVA followed by Dunnett's test.

SHXT has protective effects against myocardial infarction, as shown in Figure 1(a). There were no significant differences in the size of AAR among all groups. The vehicle group had a high proportion of infarcted tissue (INF/AAR ratio) of 44.5 ± 5.0%, which was significantly reduced by pretreatment with SHXT. Intravenous administration of SHXT at doses of 10 and 30 mg kg^−1^ resulted in INF/AAR ratios of 14.0 ± 0.2% (*P* < .01) and 6.2 ± 1.2% (*P* < .01), respectively. Oral administration of SHXT also dose-dependently reduced this ratio. Similarly, the INF/total LV ratio was also significantly lower in rats with SHXT-treatment than in vehicle-treated rats.

### 3.3. Plasma CK-MB, LDH and Troponin I

As shown in Figures 1(b)–1(d), in the sham group, all three cardiac enzymes remained at low levels at the end of surgery, indicating that a sham-operation without I/R did not result in significant myocardial injury. In the vehicle group, I/R caused increased levels of all these cardiac enzymes. However, pretreatment with SHXT by intravenous injection and oral administration both attenuated the increases of all these cardiac enzymes, in a concentration-dependent manner.

### 3.4. Myocardial DNA Fragmentation

**Figure 2:**
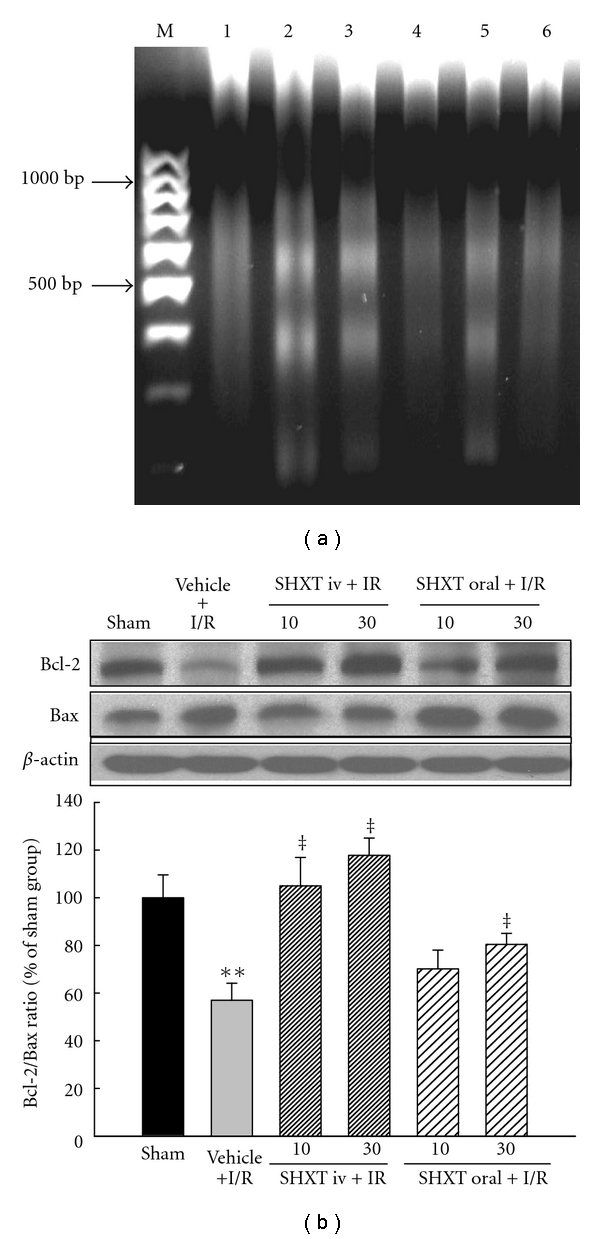
SHXT attenuated ischemia/reperfusion (I/R)-induced myocardial apoptosis by the DNA ladder and Bcl-2/Bax ratio. (a) DNA fragmentation was detected by agarose gel electrophoresis. M represents marker; lane 1 represents sham tissue; lane 2 represents vehicle-treated tissue; lane 3 represents SHXT 10 mg/kg-treated tissue (i.v.); lane 4 represents SHXT 30 mg/kg-treated tissue (i.v.); lane 5 represents SHXT 10 mg/kg-treated tissue (oral); lane 6 represents SHXT 30 mg/kg-treated tissue (oral). Similar results were obtained in four other experiments. (b) Quantitative analysis on the Bcl-2/Bax ratio by western blots. Each value represents the mean ± SEM of six rats. **P < .01 versus sham group; ^+^ *P* < .05, ^‡^ *P* < .01 versus vehicle-treated rats. ANOVA followed by Dunnett's test.

In the sham group, there was no DNA fragmentation detected in myocardial tissue (Figure 2(a), lane 1). In contrast, the formation of DNA nucleosome fragmentation was clearly detected in myocardial tissues in the vehicle group (Figure 2(a), lane 2). Both intravenous and oral administration of high dose (30 mg kg^−1^) SHXT markedly decreased myocardial DNA fragmentation (Figure 2(a), lanes 4 and 6).

### 3.5. Myocardial Expression of Bcl-2 and Bax

As shown in Figure 2(b), I/R caused decreased expression of Bcl-2 protein and increased expression of Bax protein in myocardium, resulting in a decrease of the Bcl-2/Bax ratio, indicating I/R-induced apoptosis. However, intravenous administration of SHXT prevented the decrease of the Bcl-2/Bax ratio dose-dependently. Similarly, but to a lesser extent, oral administration of SHXT at a high dose attenuated the decrease of the Bcl-2/Bax ratio.

### 3.6. Myocardial eNOS Protein and Plasma NO Production

**Figure 3:**
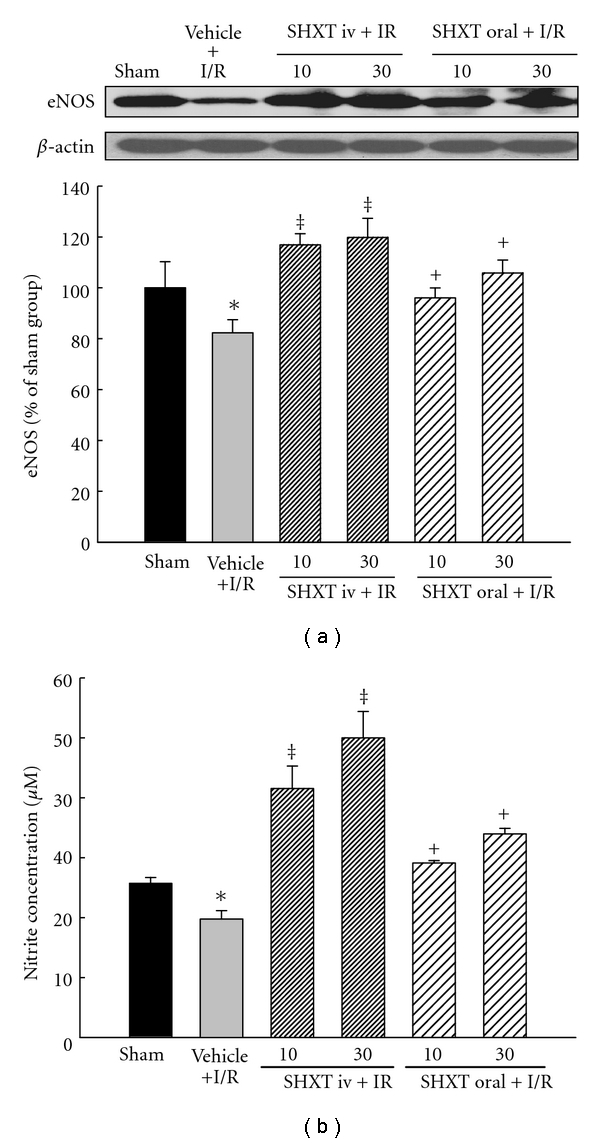
SHXT up-regulated eNOS protein expression and then promoted plasma NO production after I/R. (a) The expression level of eNOS protein in infarcted myocardial tissues was measured by immunoblot assay. The relative ratio of protein/*β*-actin protein was quantified by densitometric analyses. (b) NO production was detected spectrophotometrically by measuring its metabolite, nitrite. Each value represents the mean ± SEM of six rats. *P < .05 versus sham group; ^+^ *P* < .05, ^‡^ *P* < .01 versus vehicle-treated rats. ANOVA followed by Dunnett's test.

As shown in Figure 3(a), I/R decreased the expression of myocardial eNOS protein. However, both intravenous injection and oral administration of SHXT prevented the decrease dose-dependently. Similarly, as shown in Figure 3(b), I/R caused a decreased level of plasma nitrite, and SHXT had a significantly antagonizing effect on this change, in a dose-dependent manner.

### 3.7. MAPK Pathway

**Figure 4:**
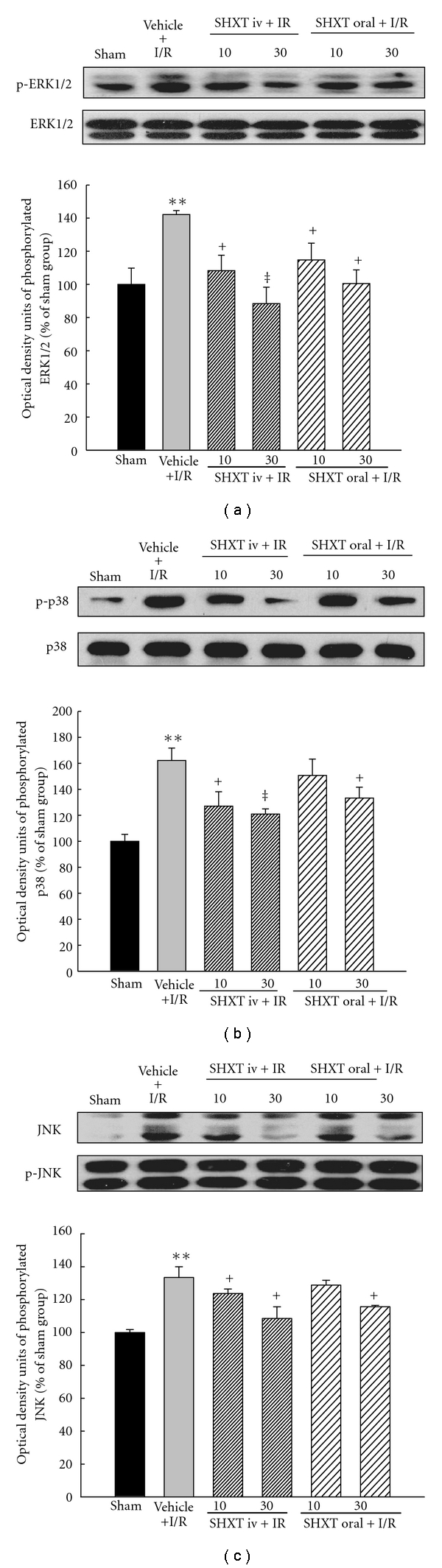
Effects of SHXT on activations of ERK1/2 (a), p38 (b) and JNK (c) induced by I/R. Each value represents the mean ± SEM of six rats. **P < .01 versus sham group; ^+^ *P* < .05, ^‡^ *P* < .01 versus vehicle-treated rats. ANOVA followed by Dunnett's test.

We finally explored the signaling pathway of SHXT-mediated anti-apoptotic effects, and examined the MAPK cascades, the upstream signaling molecules in apoptotic reactions. As shown in Figure 4(a), in comparison with the sham group, the phosphorylation of ERK1/2 was increased in the vehicle group due to I/R. However, the I/R-induced activation of ERK1/2 was significantly abolished by pretreatment with SHXT. In addition, SHXT also attenuated the activation of p38 induced by I/R (Figure 4(b)), even though this effect was not significant when a low dose (10 mg kg^−1^) was administered orally. Similarly, I/R-induced activation of JNK was attenuated by SHXT (Figure 4(c)).

## 4. Discussion

This is the first study to demonstrate that SHXT, a traditional Chinese medicinal formula, has composite cardioprotective effects against I/R of antiarrhythmia, reducing infarct size and increased survival in rats. In addition, SHXT possesses a potent inhibitory effect against I/R-induced myocardial apoptosis, which may be mediated, at least in part, by eNOS and MAPK pathways.

In our rat model with myocardial damage induced by I/R, we first found that pretreatment with SHXT significantly decreased the rhythm disturbances, mortality rate and myocardial infarct size. During an ischemic insult, myocytes may release a variety of intracellular enzymes and proteins into the blood. In the present study, we examined multiple biochemical markers including CK-MB, LDH and troponin I to detect cardiac cellular damage caused by I/R. We found that I/R-induced elevations of plasma LDH, CK-MB and troponin I were markedly blunted when rats were pretreated with SHXT.

**Figure 5:**
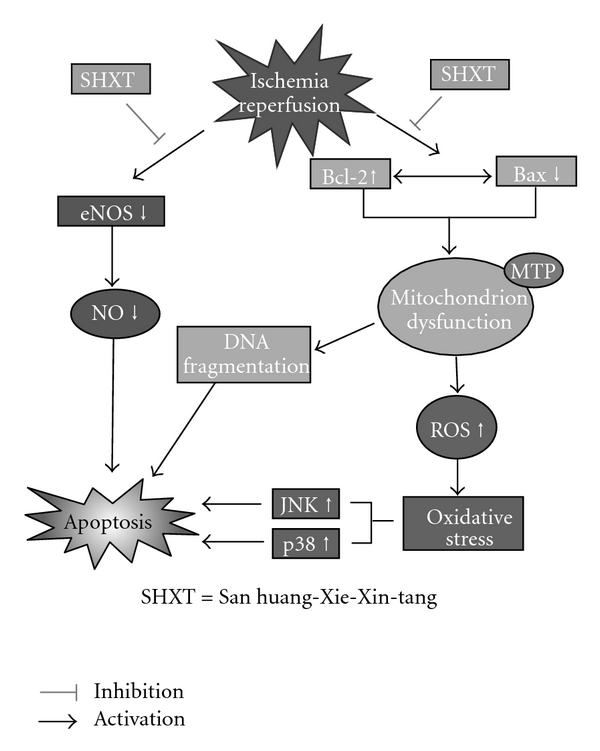
Proposed mechanisms of the cardioprotective effects of SHXT on ischemia/reperfusion-induced myocardial apoptosis according to results in the present study. SHXT can significantly inhibit myocardial apoptosis induced by ischemia/reperfusion, at least in part, by activating eNOS expression and blunting MAPK-mediated cascades.

Two distinct types of myocardium cell death, necrosis and apoptosis, have been linked with I/R-induced myocardial injury. Internucleosomal DNA fragmentation observed in agarose gels [21], chromatin condensation observed by Hoechst 33342 staining and flow cytometric analysis for translocation of phospholipid phosphatidylserine by annexin V/PI staining [22], have been widely used to define myocardial apoptosis. The mitochondria apoptotic pathway has been described as important for apoptotic cell death signaling for mammalian cells [23]. Apoptosis after I/R has been found to be associated with increased levels of Bax protein and also with a decreased Bcl-2/Bax ratio [24]. The overexpression of Bcl-2 in mice can significantly inhibit apoptosis and decrease infarct size in the heart after I/R [25]. Therefore, we investigated the role of Bcl-2 family proteins in the anti-apoptotic effects of SHXT. Our results showed that SHXT treatment significantly increased Bcl-2 expression and decreased Bax, suggesting that changes in the ratio of proapoptotic and antiapoptotic Bcl-2 family proteins may contribute to the antiapoptotic and cardioprotective effects of SHXT on I/R injury (Figure 5).

Numerous studies have demonstrated the cardioprotective effects of NO during I/R. In addition, it has been demonstrated that the effects of NO on myocardial I/R injury include both pro- and anti-apoptotic effects depending on the source of NO [26, 27]. However, precise mechanisms underlying these effects are complex and not completely understood. For example, in eNOS^−/−^ mice, myocardial injury was found to be exacerbated following I/R, suggesting the protective role of eNOS-derived NO [28, 29]. Additionally, treatment with statins has been found to reduce I/R injury after myocardial infarction, via PI3-kinase/Akt and eNOS-mediated pathways with increased NO availability [30]. Some studies suggest that the beneficial effects of NO from eNOS are mediated by regulation of vascular tone, superoxide radical scavenging and inhibition of neutrophil adherence and infiltration [27–31]. Our results were consistent with previous studies showing that the myocardial expression of the eNOS protein was markedly decreased after I/R injury. In addition, we showed that administration of SHXT increased the expression of the eNOS protein, promoted NO generation and attenuated myocardial apoptosis in the I/R animal model. Collectively, the present study suggests that SXHT may protect myocardial tissue from apoptosis by modulating eNOS expression (Figure 5). Our results are in line with a recent study showing that hydroxysafflor yellow A, a flavonoid of *Carthamus tinctorius* extracts, can inhibit I/R-induced opening of mitochondrial permeability transition pores through enhanced NO production by eNOS activation [31].

Emerging evidence suggests that MAPK-signaling cascades play an important role in oxidative stress-induced apoptotic cell death [31, 32]. The MAPK family has been classified into three major subfamilies: the extracellular signal-regulated kinase (ERK), the c-Jun N-terminal kinase (JNK) and the p38 MAPK. They are activated in response to myocardial I/R, and it is generally believed that the activations of ERK1/2 (beneficial) and p38 MAPKs-JNKs (deleterious) exert opposite effects on post-ischemic myocardial apoptosis and cardiac function recovery [33, 34]. Namely, activation of ERK contributes to cell differentiation, proliferation and survival, whereas JNK and p38 are activated by pro-inflammatory cytokines and environmental stresses and promote apoptosis [33]. However, there is some evidence suggesting that ERK1/2 also contributes to cell death in many organ systems. For example, ERK1/2 was activated in an animal model of I/R-induced brain injury, and inactivation of ERK1/2 reduced the extent of tissue damage [35, 36]. In cardiomyocytes, a recent study has also shown activation of ERK1/2 to be important in Bcl-2 family-mediated cell apoptosis caused by doxorubicin [37]. Therefore, we finally examined the potential effects of SHXT on the activation of the different MAPK cascades induced by I/R. We found that activations of ERK1/2, p38 and JNK were observed in rat hearts subjected to I/R, and pretreatment with SHXT significantly decreased the activation of these activations. Our results indicate that SHXT suppresses activations of all three subfamilies of MAPK-signaling cascades induced by myocardial I/R injury. Even though these data suggest that SHXT may inhibit the survival action of ERK1/2, we speculate that this effect may be offset by p38 and JNK, with the net effect of MAPKs potentially contributing to the anti-apoptotic action of SHXT.

In conclusion, these results demonstrate for the first time that SHXT, a Chinese herbal medicine, has potent cardioprotective effects with anti-apoptosis in rats with myocardial I/R injury, and that these antiapoptotic effects are mediated partly by eNOS and MAPK pathways (Figure 5). This study implicates that SHXT is a potentially useful drug that can be applied clinically in the prevention or treatment of myocardial injury caused by I/R.

## Figures and Tables

**Table 1:** Effect of SHXT on the severity of arrhythmias induced by ischemia/reperfusion in anesthetized rats.

Groups (*n* = 8)	Total VEBs	Duration of VT (s)	Duration of VF (s)	VT (%)	VF (%)	Irreversible VF (%)	Mortality (%)	Scores
Sham	63 ± 6	0	0	0	0	0	0	1 ± 1
I/R + vehicle	781 ± 49**	67 ± 3**	23 ± 3**	100	83	17	53	5 ± 1**
I/R + SHXT (10 mg/kg, i.v.)	326 ± 54^‡^	37 ± 8^+^	10 ± 3^+^	100	33	0	0	3 ± 1^‡^
I/R + SHXT (30 mg/kg, i.v.)	166 ± 28^‡^	19 ± 3^‡^	3 ± 1^‡^	67	33	0	0	2 ± 1^‡^
I/R + SHXT (10 mg/kg, oral)	535 ± 35^+^	42 ± 4^+^	20 ± 5	100	83	0	33	4 ± 1
I/R + SHXT (30 mg/kg, oral)	210 ± 30^‡^	21 ± 3^‡^	6 ± 1^‡^	67	33	0	0	2 ± 1^‡^

Values are mean ± SEM. Evaluation of arrhythmias described in Methods section. VEB: ventricular ectopic beats; VT: ventricular tachycardia; VF: ventricular fibrillation; I/R: ischemia/reperfusion; SHXT: San-Huang-Xie-Xin-Tang. ANOVA followed by Dunnett's test.

**P < .01 versus sham group; ^+^ *P* < .05, ^‡^ *P* < .01 versus vehicle-treated rats.
